# Development of a multi-functional chamber for resonant X-ray scattering experiments in the tender X-ray regime at the PAL-XFEL

**DOI:** 10.1107/S1600577525002899

**Published:** 2025-04-22

**Authors:** Soon Hee Park, Seonghan Kim, Jaeku Park, Seokhwan Yun, Jaehong Jeong, Je-Geun Park, Kyung Sook Kim, Tae-Kyu Choi, Intae Eom, Dogeun Jang, Minseok Kim, Jae Hyuk Lee, Sang-Youn Park, Hyunjung Kim, Sae Hwan Chun

**Affiliations:** a PAL-XFEL Division, Pohang Accelerator Laboratory, POSTECH, Pohang, Gyeongbuk37673, Republic of Korea; b Department of Physics and Astronomy Seoul National University Seoul08826 Republic of Korea; c Center for Ultrafast Phase Transformation, Department of Physics Sogang University Seoul04107 Republic of Korea; Brazilian Synchrotron Light Laboratory, Brazil

**Keywords:** tender X-rays, resonant X-ray scattering, XFEL, ruthenate, 4*d* orbital transition metal oxides

## Abstract

The hard X-ray undulator line at Pohang Accelerator Laboratory X-ray Free Electron Laser (PAL-XFEL) provides a wide range of photon energies encompassing both the tender (2–5 keV) and hard (5–15 keV) X-ray regimes. Its Femtosecond X-ray Scattering (FXS) endstation is dedicated to research in condensed matter physics and materials science, and supports various time-resolved X-ray experiments, such as diffraction, spectroscopy and resonant X-ray scattering. We report the development of a multi-functional chamber at the FXS endstation to support the experiments in the tender X-ray regime where significant air scattering and absorption pose challenges. This chamber enables optical-pump/X-ray-probe experiments in a vacuum environment, with its functionality demonstrated through time-resolved resonant elastic X-ray scattering experiments on a ruthenate Li_2_RuO_3_ near the Ru *L*_3_ (∼2.84 keV) absorption edge. Designed to readily accommodate modular instruments, the chamber offers flexibility to provide diverse experiment conditions requested by users at the FXS endstation.

## Abstract 2

A multi-functional chamber at the Femtosecond X-ray Scattering endstation of the PAL-XFEL is introduced, developed for time-resolved resonant X-ray scattering experiments in the tender X-ray regime (2–5 keV).

## 1. Introduction

Resonant X-ray scattering experiments have significantly advanced condensed matter physics by enabling the detection of ground and excited states associated with fundamental physical degrees of freedom (*i.e.* charge, spin, orbital and lattice) (Templeton & Templeton, 1982 ▸; Dmitrienko, 1983 ▸; Hill & McMorrow, 1996 ▸; Kotani & Shin, 2001 ▸; Lovesey *et al.*, 2005 ▸; Ament *et al.*, 2011 ▸; de Groot *et al.*, 2024 ▸; Mitrano *et al.*, 2024 ▸). These techniques exploit the X-ray absorption process, where core-level electrons are excited to valence states, and then measure the X-ray scattering signals from the X-ray emission process, where higher-energy electrons refill the core-level vacancies. Throughout these processes, the elastic and inelastic scattering signals, linked to the ground and excited states, respectively, are significantly enhanced, as they would otherwise be too weak to detect.

Initially established at synchrotron X-ray facilities, these methods have been employed to study various ordered states and their excitations in condensed matter systems, including spin, orbital and charge density wave orders (Hill & McMorrow, 1996 ▸; Murakami *et al.*, 2007 ▸; Ghiringhelli *et al.*, 2012 ▸). The advent of X-ray free-electron lasers (XFELs) with unprecedented brightness, long coherence lengths and atto-/femto-second scale pulse durations (Emma *et al.*, 2010 ▸; Ishikawa *et al.*, 2012 ▸; Kang *et al.*, 2017 ▸; Prat *et al.*, 2020 ▸; Decking *et al.*, 2020 ▸) has further advanced resonant X-ray scattering techniques. In particular, the XFELs enable time-resolved studies of ultrafast dynamics, capturing the evolution of ground and excited states induced by photoexcitation using optical laser pumps (Kubacka *et al.*, 2014 ▸; Först *et al.*, 2015 ▸; Dean *et al.*, 2016 ▸; Cao *et al.*, 2019 ▸; Jang *et al.*, 2023 ▸).

While the resonant X-ray scattering experiments have been conducted extensively in the soft X-ray (0.1–2 keV) and hard X-ray (5–100 keV) regimes, the photon energy range of 2–5 keV, known as the ‘tender X-ray regime’, remains under-utilized, primarily due to the limited number of available facilities worldwide. This energy range holds particular significance for quantum materials research, as it encompasses the *L*-absorption edges of 4*d*-orbital transition-metal elements. These elements underpin many intriguing quantum materials that exhibit a wide array of quantum mechanical phenomena, including triplet superconductivity, orbital order, Haldane chain, quantum spin liquid, Higgs mode and altermagnetism (Ishida *et al.*, 1998 ▸; Khalifah *et al.*, 2002 ▸; Lee *et al.*, 2006 ▸; Banerjee *et al.*, 2017 ▸; Do *et al.*, 2017 ▸; Jain *et al.*, 2017 ▸; Zhu *et al.*, 2019 ▸).

In response to growing interest, many X-ray radiation facilities are being upgraded or newly constructed to provide access to the tender X-rays (Suzuki *et al.*, 2019 ▸; Abraham *et al.*, 2019 ▸; Ditter *et al.*, 2020 ▸; Gretarsson *et al.*, 2020 ▸; Rovezzi *et al.*, 2020 ▸; Mankowsky *et al.*, 2021 ▸; Ismail *et al.*, 2024 ▸; Peredkov *et al.*, 2024 ▸). Resonant X-ray scattering experiments in this regime require carefully designed instrumentation, including vacuum chambers to reduce substantial X-ray absorption and scattering caused by ambient air. In addition, repurposing instruments originally designed for the soft and hard X-ray regimes offers a cost-effective approach to expanding experimental capabilities into the tender X-ray regime.

In this work, we present the design of a multi-functional chamber enabling time-resolved X-ray diffraction, spectroscopy and resonant X-ray scattering at the Femtosecond X-ray Scattering (FXS) endstation of the hard X-ray Scattering and Spectroscopy (XSS) beamline at the PAL-XFEL (Park *et al.*, 2016 ▸; Eom *et al.*, 2022 ▸; Choi *et al.*, 2023 ▸). The chamber is designed to integrate with a two-circle (θ and 2θ) diffractometer in the horizontal scattering geometry, a two-dimensional (2D) JUNGFRAU detector, and a data acquisition (DAQ) system, all of which are already available at the beamline (Park *et al.*, 2016 ▸; Eom *et al.*, 2022 ▸; Park *et al.*, 2019 ▸; Mozzanica *et al.*, 2014 ▸). We validate the chamber’s functionality by performing time-resolved resonant elastic X-ray scattering (REXS) experiments on a bulk single-crystal ruthenate, Li_2_RuO_3_, near the Ru *L*_3_ absorption edge. The experimental capabilities of this setup can be readily expanded with additional modular instruments, enabling diverse experiment conditions required for user experiments at the FXS endstation.

## 2. Instrumentation

### 2.1. Key X-ray optics at the optical hutch and XSS beamline of the hard X-ray undulator line

**Figure 1:**
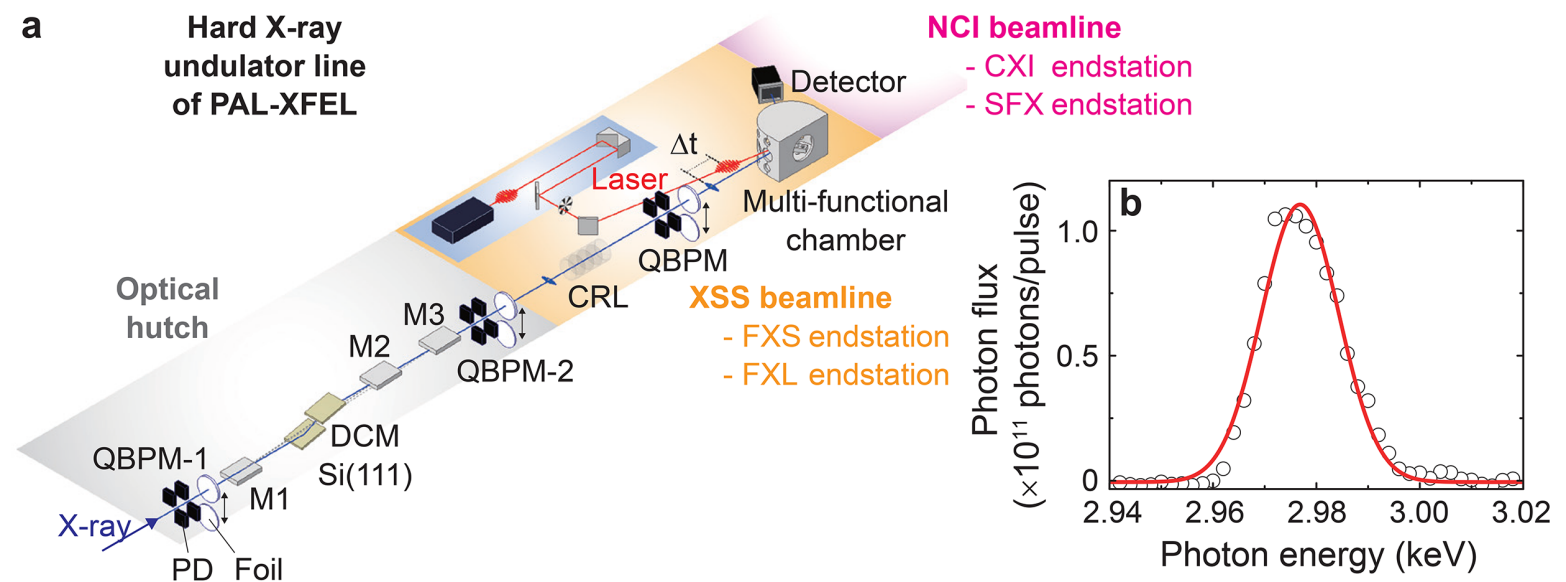
(*a*) Schematic layout of the optical hutch (OH, gray) and the XSS beamline (orange). The primary components in the OH, from upstream to downstream, include QBPM-1 [comprising photodiodes (PD) and changeable foils], the M1 mirror, the Si(111) DCM, the M2 and M3 mirrors, and QBPM-2. The X-ray beam (blue line) is focused by a Be-CRL, passes through a QBPM, and is delivered to the multi-functional chamber at the FXS endstation of the XSS beamline. For time-resolved experiments, the optical laser pump, manipulated within a closure system (blue), is directed into the chamber. (*b*) Energy profile of the pink beam centered at *E*_i_ = 2.977 keV. The data points (symbols) are fitted with a Gaussian function (red line), yielding a full width at half-maximum (FWHM) of 18 eV.

The hard X-ray undulator line at PAL-XFEL generates X-rays with photon energies ranging from 2 to 20 keV (refer to Table 1 ▸ for details). It consists of an optical hutch and two experimental hutches arranged in tandem: the upstream XSS beamline and the downstream Nano Crystallography and Imaging (NCI) beamline [Fig. 1 ▸(*a*)] (Eom *et al.*, 2022 ▸). The XSS beamline houses two endstations, Femtosecond X-ray Scattering (FXS) and Femtosecond X-ray Liquidography (FXL), while the NCI beamline hosts two endstations, Coherent X-ray Imaging (CXI) and Serial Femtosecond Crystallography (SFX).

Fig. 1 ▸(*a*) illustrates the layout of the optical hutch (OH) and the XSS beamline. The main X-ray optical components in the OH include X-ray mirrors (M1, M2 and M3), a silicon double-crystal monochromator [Si(111) DCM], and quadrant beam position monitors (QBPMs). The mirrors, made of Si single crystals with carbon coating, are designed to suppress the higher harmonics of X-rays through total reflection. M1, positioned upstream of the DCM, moves upward to reflect the X-ray beam with a variable pitch angle ranging from 1.4 to 3 mrad. M2 and M3, located downstream of the DCM, move downward to reflect the beam with a vertical beam offset of 30 mm, maintaining fixed pitch angles of 3 mrad and 1.4 mrad, respectively. These pitch angles determine the choice of mirror combinations: M1 and M2 for the X-ray photon energy range 2–9.8 keV and M1 and M3 for 9.8–20 keV. For monochromatic beam conditions, the mirrors are bypassed (or moved in the opposite directions), allowing X-rays to pass directly to the DCM.

Fig. 1 ▸(*b*) shows a representative energy profile of the pink beam, generated through the self-amplified spontaneous emission (SASE) process in the hard X-ray undulator line at the PAL-XFEL. This profile, centered at *E*_i_ = 2.977 keV, was recorded using the signal from the downstream QBPM [QBPM-2 in the OH; see Fig. 1 ▸(*a*)], averaged over 60 pulses at each energy while scanning the Si(111) DCM. This pink beam delivers ∼1.5 mJ pulse^−1^ with a bandwidth of 18 eV (FWHM, Δ*E*/*E*_i_ ≃ 6 × 10^−3^) (Kang *et al.*, 2017 ▸), and its profile remains consistent in a wide X-ray photon energy window of 2–15 keV. For higher X-ray photon energies, up to 20 keV, the pulse energy decreases to ∼1 mJ pulse^−1^, while the bandwidth is preserved.

The monochromatic beam is prepared using Si(111) or higher-order reflections of the DCM. Its bandwidth is determined by the Darwin width of the Si single crystals. For instance, the Darwin width of the Si(111) DCM at*E*_i_ = 2.98 keV corresponds to Δ*E* ≃ 0.39 eV. Although the photon flux decreases from ∼3.14 × 10^12^ photons pulse^−1^ (pink beam condition) to ∼1.05 × 10^11^ photons pulse^−1^ for the monochromatic beam – and is further reduced after passing through diagnostic tools such as the QBPMs down to a few 10^10^ photons pulse^−1^ – it remains sufficient for conducting X-ray spectroscopy and resonant X-ray scattering experiments.

The QBPMs consist of four photodiodes that measure the back-scattered signal from an X-ray-transparent foil placed downstream of the beam. Depending on the X-ray photon energy, one of three foils, *i.e.* 0.5 µm- or 2 µm-thick Si_3_N_4_ films or a 20 µm-thick CVD-grown diamond crystal, can be selected based on X-ray transmittance and back-scattering angle. Two pairs of photodiodes, positioned horizontally and vertically, monitor the respective positions of the X-ray beam (Tono *et al.*, 2011 ▸). The photodiode signals are captured by a four-channel digitizer with timestamp tagging. The relative ratios of the horizontal and vertical photodiode signals are used to calibrate the beam position on a pulse-to-pulse basis, enabling estimation of beam-pointing jitter to less than 7.8% of the beam size at 40 m downstream from the last undulator (Kang *et al.*, 2017 ▸). In addition, the sum of their signals, *i.e.* the back-scattered signal from the foil, is recorded for each X-ray pulse and can be used for normalizing the sample signals on a per-pulse basis. In the OH, QBPM-1 and -2 are positioned upstream and downstream of the mirrors and the DCM, respectively. Another QBPM is located at the XSS beamline and is used to monitor the X-ray pulse intensity and position near the sample.

The XSS beamline employs beryllium compound refractive lenses (Be-CRLs) to focus the X-ray beam. Multiple stacks of Be-CRLs are housed in a chamber, with one selected based on the desired X-ray beam size. For tender X-rays at *E*_i_ ≃ 3 keV, a Be-CRL (diameter: 500 µm, radius of curvature: 1000 µm, thickness: 300 µm) produces a focused X-ray beam size of ∼200 µm (FWHM) at the sample position and a transmittance of ∼30%. In the higher X-ray photon energy regime, the beam can be further focused to a diameter >3 µm (FWHM) by using other stacks of Be-CRLs.

### 2.2. Multi-functional chamber design

**Figure 2:**
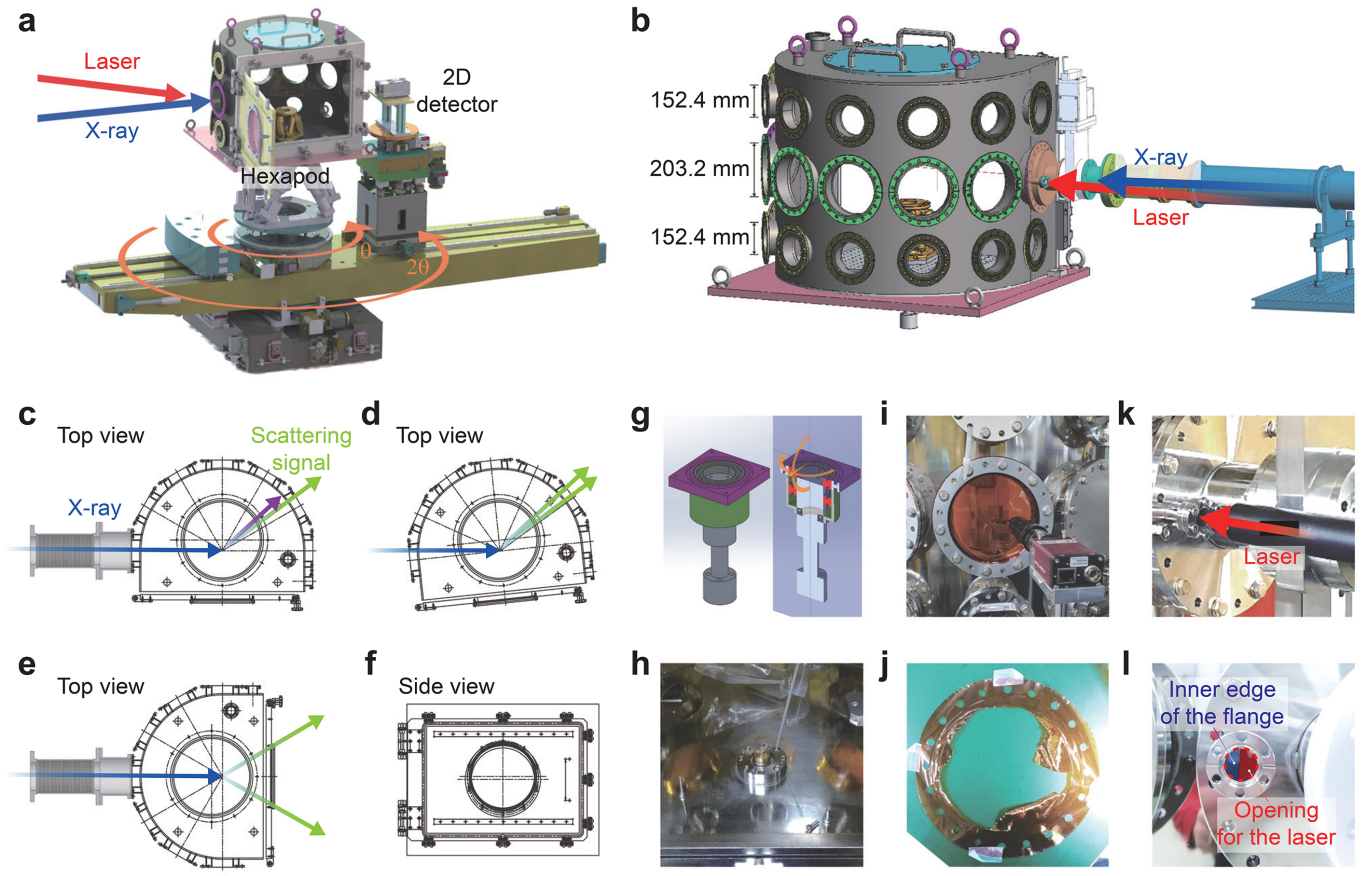
(*a*) Schematic illustration of the experimental geometry using the multi-functional chamber mounted on the heavy-load two-circle diffractometer at the FXS endstation. The blue and red arrows indicate incident X-ray and laser beams, respectively, while the orange arrows represent the motions of θ and 2θ rotational stages. (*b*) Side view of the chamber connected to a vacuum pipe from the XSS beamline. The X-ray beam (blue arrow) enters the chamber through the vacuum pipe, and the laser beam (red arrow) is delivered through a 25.4 mm-diameter quartz window. (*c*, *d*) Top views of the chamber configurations: (*c*) without θ-rotation and (*d*) with θ-rotation of the diffractometer. The green arrows indicate diffracted/scattered X-ray beams exiting through the 203.2 mm-diameter chamber windows, while the purple arrow denotes the beam blocked by the chamber or ports. (*e*, *f*) Top view (*e*) and side view (*f*) of the experimental setup in a transmission WAXS geometry. (*g*) Schematic of a cylindrical pole sealing the 56 mm-diameter circular opening located at the bottom of the chamber, secured with O-rings and supported by ball bearings for vacuum integrity. (*h*) Photograph of the cylindrical pole assembly. (*i*) A 140 µm-thick Kapton film window installed on the chamber. (*j*) Example of a punctured 50 µm-thick Kapton film due to air pressure during the chamber pumping process. (*k*, *l*) Quartz optical window used for laser beam incidence. The optical laser beam [red arrow in (*k*)] passes through the unobstructed portion of the opening, avoiding the flange’s inner edge (*l*).

We designed a multi-functional chamber to be integrated with a two-circle diffractometer at the FXS endstation, as shown in Fig. 2 ▸(*a*). The chamber has a roughly half-cylindrical shape, with an inner radius of 375 mm and a height of 580 mm [Fig. 2 ▸(*b*)]. Its curved side features three rows of ports: in each row, individual ports are spaced 30° apart, and all ports are sealed with O-rings to maintain a vacuum down to ∼10^−5^ Torr. The middle row contains the 203.2 mm-diameter ports for incident and diffracted (scattered) X-rays, while the remaining 152.4 mm-diameter ports are used for connection of cables and vacuum pump lines. The flat side [Fig. 2 ▸(*f*)] of the chamber includes a large, hinged door (606 mm × 424 mm) with O-ring vacuum sealing and provide easy access to the inside.

This chamber is supported by a large hexapod positioned atop the diffractometer’s θ-rotational stage, enabling positional adjustments via either the hexapod or the θ-rotational stage. For instance, blind spots caused by port locations on the chamber can be mitigated by tilting or rotating the chamber within an angular range of ±6° [Figs. 2 ▸(*c*) and 2(*d*)]. In turn, the accessible 2θ angle covers the ranges 0–12°, 18–42°, 48–72° and 78–102°, while the χ angle spans a range of ±15°. (No azimuth angle is available.) Incident X-rays are delivered to the sample through a vacuum pipe connecting the spool end to the chamber [Fig. 2 ▸(*b*)]. The X-rays diffracted (or scattered) from the sample exit the chamber through 203.2 mm-diameter ports in the horizontal scattering geometry. These signals are captured by a 2D detector placed on the diffractometer’s 2θ arm stage. The 203.2 mm-diameter windows are made of a polyimide (*e.g.* Kapton) film with an optimal thickness of ∼140 µm [Fig. 2 ▸(*i*)]. We found that using the proper film thickness is crucial; a thinner film cannot withstand external pressure and risks puncturing [Fig. 2 ▸(*j*)], while a thicker film significantly reduces the transmitted signal. Alternatively, beryllium windows can be used, as they offer minimal transmission loss compared with the Kapton film. For optical laser pumps, a 25.4 mm-diameter quartz optical window port is incorporated into the chamber, allowing transmission of laser beams with wavelengths ranging from 240 nm to 2600 nm [Fig. 2 ▸(*k*) and 2(*l*)]. The laser beam illuminates the sample at 10° angle horizontally relative to the X-ray beam.

When the chamber is horizontally rotated to θ = 90°, WAXS (wide angle X-ray scattering) experiments are available in the transmission geometry [Fig. 2 ▸(*e*)]. A 238 mm-diameter glass window at the center of the chamber’s flat side door can be replaced with Kapton film, allowing the WAXS signals to reach a large 2D detector. The minimum SDD (sample-to-detector distance) is currently 100 mm but can be reduced further by redesigning the door. For this WAXS configuration with the SDD and the glass window diameter, the accessible 2θ range extends up to 50°, corresponding, for example, to *q* = 1.284 Å^−1^ at a photon energy of 3 keV.

The chamber’s top features a large 350 mm-diameter circular opening designed to accommodate additional equipment, such as a low-temperature cryostat [Figs. 2 ▸(*a*) and 2(*b*)]. At the bottom, a 56 mm-diameter circular opening houses a cylindrical pole sealed with O-rings and supported by ball bearings [Figs. 2 ▸(*g*) and 2(*h*)]. This pole transmits the horizontal rotational motion of a stage mounted on the large hexapod to the sample stage (a vacuum-compatible small hexapod or a combination of *XYZ* linear motor stages) inside the chamber. This design ensures vacuum integrity while providing effective θ-rotational motion (θ_Sample_) for the sample, independent of the diffractometer’s θ-rotational stage.

### 2.3. Time-resolved X-ray diffraction experiment in the tender X-ray regime

**Figure 3:**
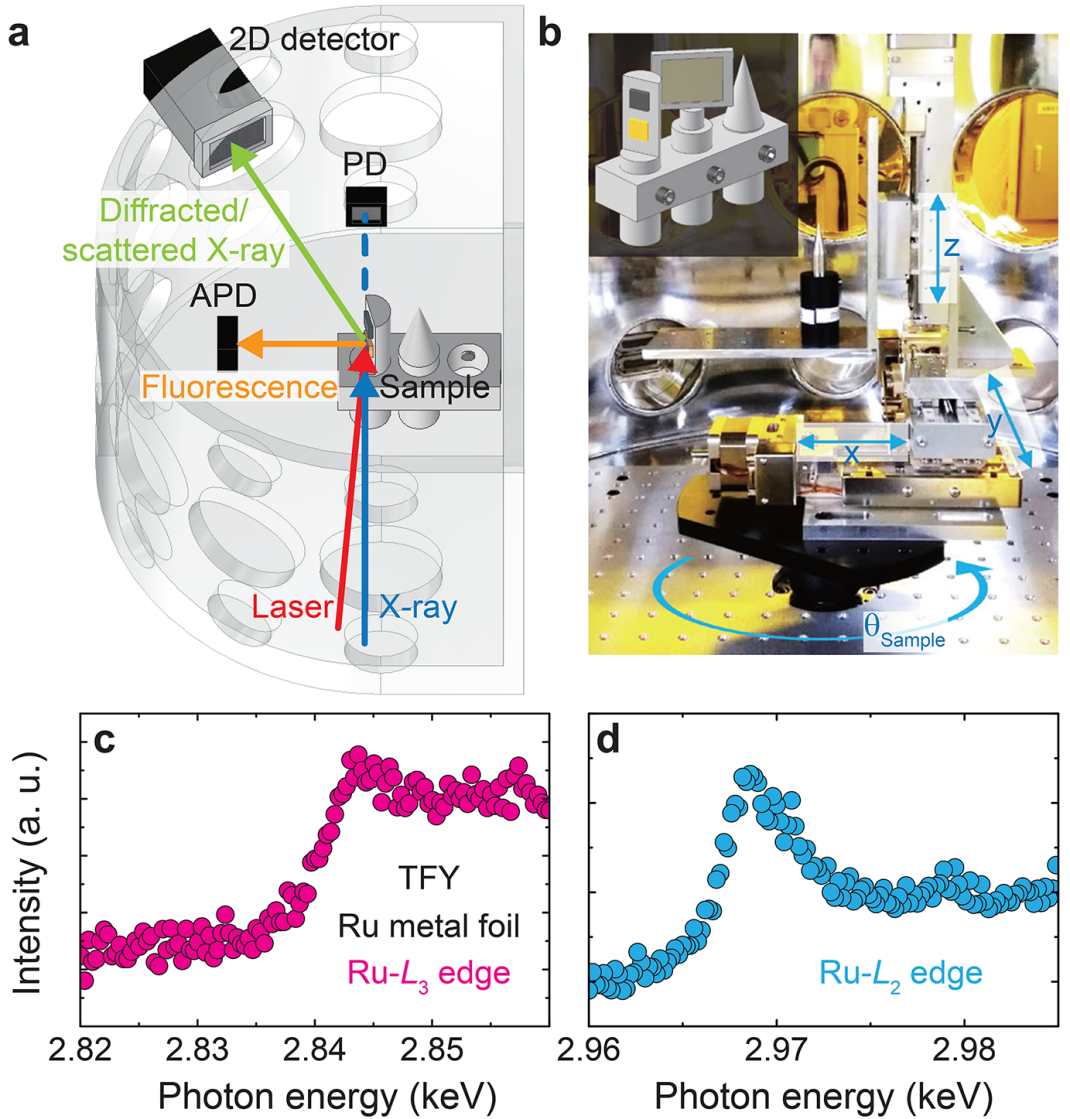
(*a*) Schematic illustration of the X-ray diffraction and X-ray absorption spectroscopy setup, including the sample stage, PD, APD and external 2D detector. The incident X-ray and laser beams on the sample are indicated by blue and red arrows, respectively. Fluorescence and diffracted/scattered beams directed to the APD and 2D detector are shown by orange and green arrows. The dotted line represents the transmitted X-ray beam path. (*b*) Photograph of the sample stage, comprising three translational stages (*x*, *y* and *z* motions) mounted on a rotational motorized stage (θ_Sample_). Inset: schematic of the sample holder assembly designed to accommodate multiple samples. X-ray absorption spectra of a Ru metal foil across the Ru *L*_3_-edge (*c*) and the Ru *L*_2_-edge (*d*), detected by the APD using total fluence yield (TFY).

We tested the feasibility of a time-resolved X-ray diffraction experiment using the multi-functional chamber under vacuum conditions [Fig. 3 ▸(*a*)]. To calibrate the X-ray photon energies, X-ray absorption spectra were obtained from a ruthenium (Ru) metal foil by scanning the monochromatic beam energy across the Ru *L*_3_ and *L*_2_ absorption edges at 2.84 keV and 2.97 keV, respectively [Figs. 3 ▸(*c*) and 3(*d*)]. An APD (avalanche photodiode) was positioned at a horizontal 2θ angle of 90°, adjacent to the foil inside the chamber. This configuration minimizes elastic X-ray scattering signals and optimizes the detection of total fluorescence emitted from the sample. Although spectral profiles measured in total-fluorescence-yield mode are prone to distortions, primarily due to self-absorption effects in concentrated samples, the features of X-ray absorption edges remain reliable for calibrating X-ray photon energies.

The APD was mounted on an optical breadboard (450 mm × 360 mm × 15 mm) installed at the bottom of the chamber. This breadboard provides a versatile platform for integrating additional equipment near the sample stage [Figs. 3 ▸(*a*) and 3(*b*)]. For example, a photodiode (PD) was placed downstream of the sample stage to assist with precise alignment and sample half-cut positioning. Both APD and PD were connected through vacuum feedthroughs to a digitizer with time stamping. Their signals were normalized using the diagnostic QBPM signals to ensure accurate measurements.

Multiple samples were mounted simultaneously on the motorized sample stage to improve efficiency by minimizing the frequent process of pumping and venting the chamber for sample exchange [Fig. 3 ▸(*b*)]. A gallium arsenide (GaAs) single crystal was used to ensure spatial overlap between the optical laser and X-ray beams, as it emits fluorescence when excited by either beam. We find that GaAs can be used not only at a wavelength of 800 nm but also across a broader range, from 240 nm to 2.6 µm. A high-magnification camera positioned outside the chamber observes the sample surface through a 203.2 mm-diameter Kapton film window, allowing precise alignment of the interaction point at the center of sample rotation.

The optical laser pump system at the XSS beamline consists of a Ti:sapphire oscillator, a regenerative amplifier and a single-pass amplifier (Kim *et al.*, 2019 ▸). The laser pulses produced by this system have Fourier-transform-limited characteristics, resulting in a short pulse duration of ∼40 fs. The fundamental wavelength of the laser is 800 nm, and it delivers a maximum pulse energy of 10 mJ at a repetition rate of 120 Hz. High harmonic generation and an optical parametric amplifier (OPA) are used to tune the frequency to cover a wavelength range of 240–2600 nm. Given that the maximum repetition rate of the X-ray at PAL-XFEL is 60 Hz, a laser chopper is employed to synchronize the laser repetition rate to either match or halve the X-ray repetition rate. This enables measurements of either laser-on behavior or alternating laser-on/off behavior at a time delay, depending on the sensitivity of the probe signal. Optical laser parameters are listed in Table 2 ▸.

**Figure 4:**
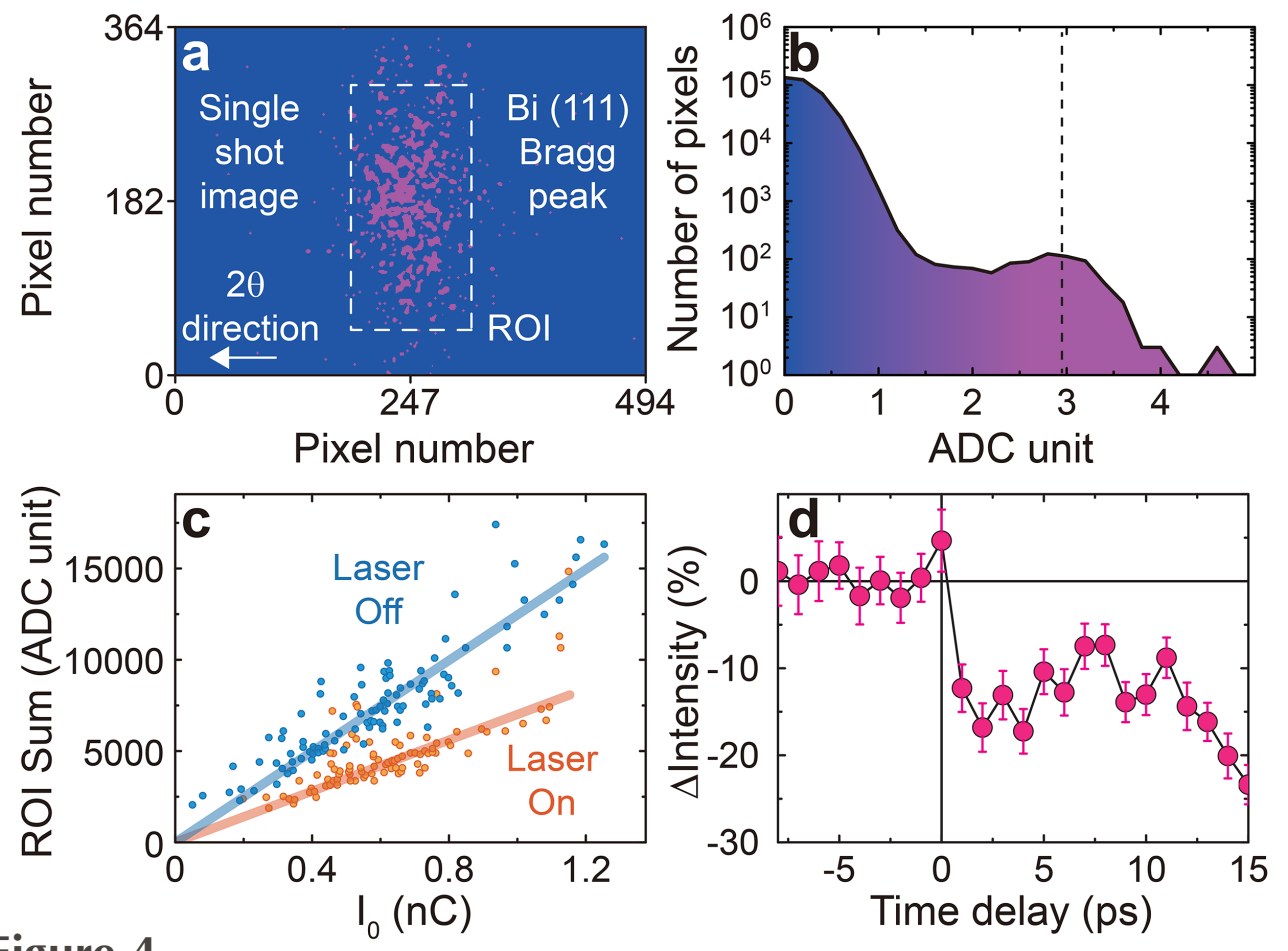
(*a*) 2D image of the (111) Bragg reflection (purple pixels) diffracted by a Bi thin film, with the region of interest (ROI) outlined by a dashed square. The white arrow indicates the increasing 2θ direction. (*b*) Histogram of the ADC units derived from the 2D image shown in (*a*). Each ADC unit corresponds to the conversion of 1 keV. (*c*) Correlation analysis showing the linear relationship between *I*_0_ (QBPM signal) and the ROI sum (2D detector). Orange and blue circles represent raw data with and without laser pumping, respectively, while solid lines show the corresponding linear fits. (*d*) Transient intensity changes of the (111) Bragg reflection, confirming the time-zero point. The error bar is derived from the standard deviation from the linear fits in the correlation analysis.

To establish temporal overlap, a bis­muth (Bi) thin film was used to measure the intensity of the (111) Bragg reflection, which changes sensitively as a function of time delay following photoirradiation by the optical laser pump (Kang *et al.*, 2017 ▸). Fig. 4 ▸(*a*) shows a single-shot image of the Bragg reflection captured using a JUNGFRAU 0.5M detector with 1024 × 512 pixels, each with a size of 75 µm × 75 µm. This detector resolves single photon energies and provides a histogram of the number of pixels versus ADC (analog-to-digital converter) units [Fig. 4 ▸(*b*)]. The ADC units are calibrated to correspond to the X-ray photon energy in keV. For instance, the data point on the dashed line in Fig. 4 ▸(*b*) indicates that ∼10^2^ pixels on the detector measure 2.968 ADC units (equivalent to 2.968 keV) for an incident X-ray pulse. This corresponds to a Bragg reflection intensity of ∼10^2^ photons pulse^−1^ or ∼6.0 × 10^3^ photons s^−1^ at a repetition rate of 60 Hz at the PAL-XFEL.

A correlation analysis of the Bragg reflection intensity is essential for capturing the transient intensity changes with high sensitivity [Fig. 4 ▸(*c*)]. Due to the stochastic nature of the SASE process, the flux of incident X-rays can vary by up to ∼90% on a pulse-to-pulse basis (Kang *et al.*, 2017 ▸). The correlation analysis provides significantly smaller error bars compared with simple averaging of X-ray diffraction signals. Fig. 4 ▸(*c*) shows a correlation plot of the X-ray diffraction signals as a function of the QBPM intensities with identical time stamps. The slope of the correlation plot is proportional to the sample signal and corresponds to a data point at a specific time delay. The standard deviation from the linear fit of the correlation plot determines the error bar for each data point. To achieve sufficient statistical accuracy for each data point, a few hundred X-ray pulses are used to construct the correlation plot. When the optical laser pump is incident, the slope of the linear relation decreases, as shown in Fig. 4 ▸(*c*). Each slope at a fixed time delay reflects the transient change of the (111) Bragg reflection intensity exhibiting an instantaneous drop at time zero, as shown in Fig. 4 ▸(*d*). We do not incorporate an arrival timing tool for the tender X-ray experiments. The temporal jitter of the X-rays at PAL-XFEL is less than 50 fs (FHWM) (Kang *et al.*, 2017 ▸), enabling us to resolve transient features on timescales beyond this without the use of a timing tool.

### 2.4. Time-resolved resonant X-ray scattering experiment in the tender X-ray regime

**Figure 5:**
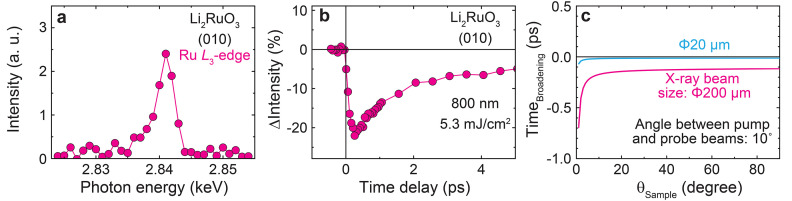
(*a*) X-ray photon energy dependence of the (010) ATS reflection of Li_2_RuO_3_ across the Ru *L*_3_-edge. (*b*) Transient intensity changes of the (010) ATS reflection at *E*_i_ = 2.841 keV. The optical laser was set to a wavelength of 800 nm with *p*-polarization, and the X-ray beam had π-polarization within the X-ray scattering plane. (*c*) Calculated temporal broadening effect as a function of θ_Sample_, resulting from the 10° incident angle difference between the pump and probe beams. The pink and cyan curves represent results for X-ray beam size diameters of 200 µm and 20 µm (FWHM), respectively.

The multi-functional chamber was used to commission time-resolved resonant X-ray scattering experiments in the tender X-ray regime. We tested the setup with a honeycomb lattice ruthenate, Li_2_RuO_3_. This material undergoes a structural phase transition below 540 K, leading to the formation of a herringbone-type dimerization of Ru—Ru bonds (Miura *et al.*, 2007 ▸; Yun *et al.*, 2019 ▸). This transition induces ATS (anisotropic tensor susceptibility) scattering at structurally forbidden momentum positions, *Q*_ATS_ = (0*k*0) (*k*: odd integer) (Dmitrienko, 1983 ▸). Fig. 5 ▸(*a*) shows the intensity profile at (010) as a function of X-ray photon energy across the Ru *L*_3_-edge. The X-ray scattering signals from the ATS reflection resonate strongly at the Ru *L*_3_-edge, enhancing the otherwise undetectable intensity. Upon excitation with an optical laser pump at a wavelength of 800 nm, the intensity of the (010) reflection drops instantaneously within 300 fs, demonstrating the system’s response to photoexcitation [Fig. 5 ▸(*b*)]. It is worth noting that the 10° angle between the optical laser pump and X-ray probe causes a temporal broadening effect, thereby degrading the temporal resolution. Fig. 5 ▸(*c*) presents the calculated temporal broadening effect as a function of θ_Sample_. For an X-ray beam size diameter of 200 µm, the effect is 0.15 ps for the (010) reflection at θ_Sample_ = 14.4° and 0.13 ps for the (111) reflection of Bi at θ_Sample_ = 33° (Fig. 4 ▸).

### 2.5. Low-temperature cryostat integrating with the multi-functional chamber

Studying quantum materials often requires cryogenic experiments to investigate various orderings that emerge at low temperatures. The FXS endstation has used a cryo-stream instrument (N-Helix, Oxford Cryosystems Ltd), which directs cryogenic gas onto the sample in ambient conditions, facilitating the optical laser beam’s incidence on the sample. However, this setup restricts the sample size to less than 1 mm × 1 mm to fit within the stream diameter and often leads to ice formation on the sample or its holder outside the stream. In addition, it cannot be used for experiments requiring vacuum conditions, such as those in the tender X-ray regime.

**Figure 6:**
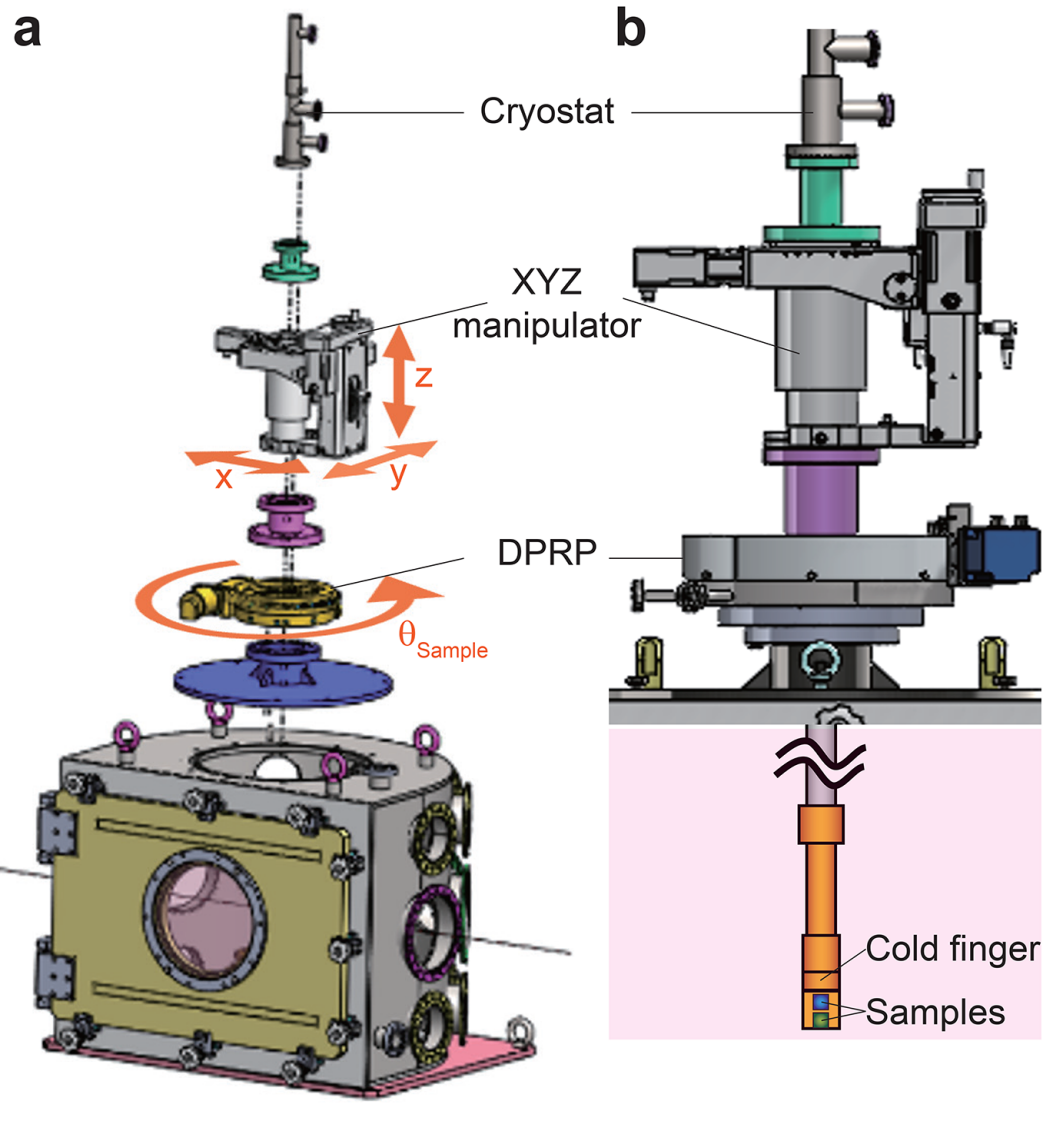
(*a*) Schematic of the chamber integrated with a cryostat, featuring a DPRP, an *XYZ* manipulator, and adapters for cryostat assembly on the upper section of the chamber. (*b*) Schematic of the upper section assembly, including the sample holder (cold finger) positioned inside the chamber.

To overcome these limitations, we employ a cryostat module designed to integrate with the multi-functional chamber (Fig. 6 ▸). The chamber’s 350 mm-diameter circular opening at the top accommodates a liquid nitro­gen or helium-flow cryostat mounted on an *XYZ* manipulator, which is installed on a differentially pumped rotatable platform (DPRP). The DPRP enables horizontal θ-motion for the sample attached to the cryostat’s cold finger. The *XYZ* manipulator positions the sample at the center of the rotation, ensuring alignment with the interaction point between the optical laser pump and X-ray probe beams. The precision of the motors is provided in Table 3 ▸. A pre-test of the base temperature using flowing liquid nitro­gen confirmed a base temperature of 77 K. While the current base pressure of ∼10^−5^ Torr for the chamber is at a low vacuum level for the low-temperature experiment, we observed that no significant ice was formed on the cold finger of the cryostat over 8 h while cooling it to and maintaining it at the base temperature. There is still room to improve the vacuum level by reducing the number of Kapton windows, which are the primary source of leaks, and by directly connecting a vacuum-compatible JUNGFRAU detector. A test with flowing liquid helium and the design of radiation shields compatible with time-resolved X-ray diffraction will be conducted at a later stage, following confirmation of the vacuum condition.

## 3. Conclusion and outlook

We introduced the design and development of a multi-functional chamber tailored for various X-ray experiments requiring vacuum conditions. This chamber was integrated with the existing equipment and infrastructure of the XSS beamline of the PAL-XFEL, including a heavy-load two-circle diffractometer, a 2D JUNGFRAU detector and a customized DAQ system. Using this integrated setup, we successfully commissioned time-resolved resonant X-ray scattering experiments in the tender X-ray regime, focusing on a ruthenate single crystal of Li_2_RuO_3_ at the Ru *L*_3_-edge. Although the commissioning experiments were conducted at room temperature, the chamber is designed to accommodate a modular cryostat, enabling studies at cryogenic temperatures. The development of this multi-functional chamber significantly enhances the capabilities of the FXS endstation, facilitating advanced investigations under diverse experimental conditions.

## Figures and Tables

**Table 1:** X-ray parameters of the hard X-ray undulator line at PAL-XFEL

Operation mode	Self-amplified spontaneous emission (SASE)	Self-seeding
Photon energy	2–20 keV	3.5–14.6 keV
Pulse energy	1–1.5 mJ pulse^−1^	1 mJ pulse^−1^
Maximum repetition rate	60 Hz	60 Hz
Bandwidth	∼20 eV	0.2–1 eV

**Table 2:** Optical laser parameters of the hard X-ray undulator line at PAL-XFEL

Center wavelength	800 nm			
Pulse duration at 800 nm (FWHM)	40 fs			
Maximum pulse energy at sample positions	<3.0 mJ at 800 nm	<0.5 mJ at 400 nm	<0.2 mJ at 266 nm	<0.03 mJ at 240–2600 nm using OPA
Repetition rate	15, 30, 60, 120 Hz			

**Table 3:** Precision of the motors for the X-ray diffractometer

	Motor		Travel range	Resolution
Room temperature setup	Diffractometer	θ	±180°	0.001°
		2θ	±90°	0.001°
		θ_Sample_	±180°	0.001°
	Sample translational stage	*x, y, z*	±25 mm	1 µm
Cryogenic temperature setup	DPRP	θ_Sample_	±180°	0.05°
	Sample translational stage	*x, y*	±12.5 mm	1 µm
		*z*	±25 mm	2 µm
